# P-1414. An Analysis of Social and Clinical Characteristics of Individuals who Acquired a Central Line Associated Bloodstream Infection (CLABSI)

**DOI:** 10.1093/ofid/ofae631.1589

**Published:** 2025-01-29

**Authors:** Derek D Schocken, Mindy Kim, Erin Gettler, Nwora Lance Okeke, Nicholas A Turner, Deverick J Anderson

**Affiliations:** Duke University School of Medicine, Durham, North Carolina; Duke University School of Medicine, Durham, North Carolina; Duke University Medical Center, Durham, NC; Duke University, Durham, NC; Duke University Medical Center, Durham, NC; Duke Center for Antimicrobial Stewardship and Infection Prevention, Durham, NC

## Abstract

**Background:**

A previous exploratory analysis of surveillance data at our large academic medical center identified higher CLABSI rates among non-Hispanic Black patients compared to White patients. In the context of a growing understanding of the importance of social determinants of health (SDOH), we sought to better describe those patients who acquired a CLABSI.

**Figure:**
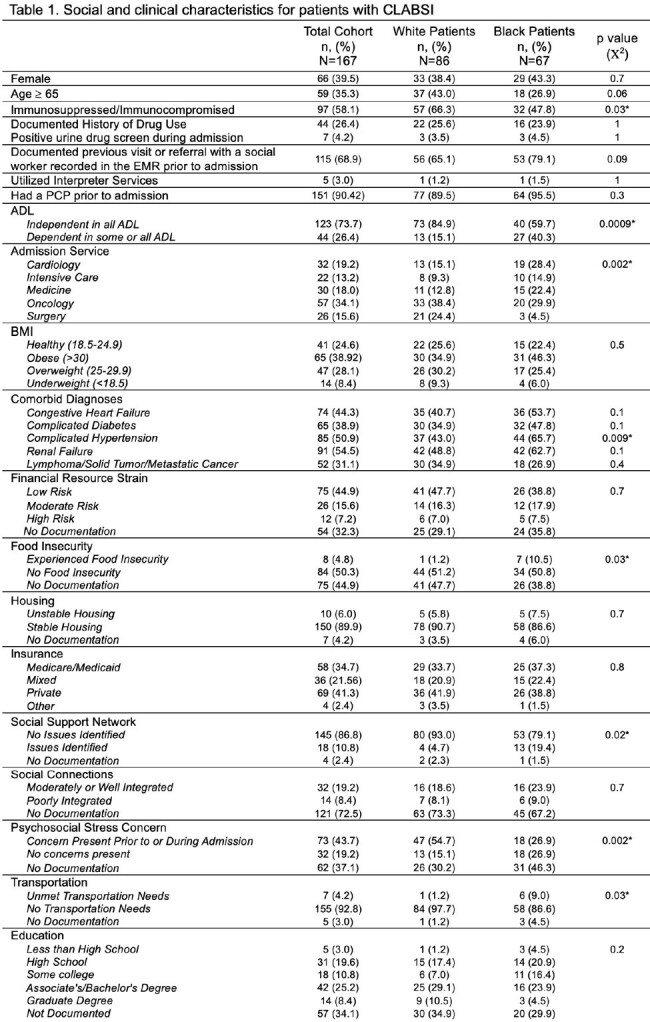

**Methods:**

We conducted a retrospective analysis of patients with CLABSI who were admitted to one of 3 hospitals in our health system in 2022 (1 large academic and 2 community hospitals). CLABSI was defined using standard NHSN criteria. We compared demographic, admission, clinical and SDOH data between patients identifying as White or non-Hispanic Black. These groups were selected due to sample size limitations. Race was defined based on information from the EHR. To identify variables associated with race that may mediate differences in acquiring CLABSI, we created a logistic regression model using stepwise backwards selection.

**Figure:**
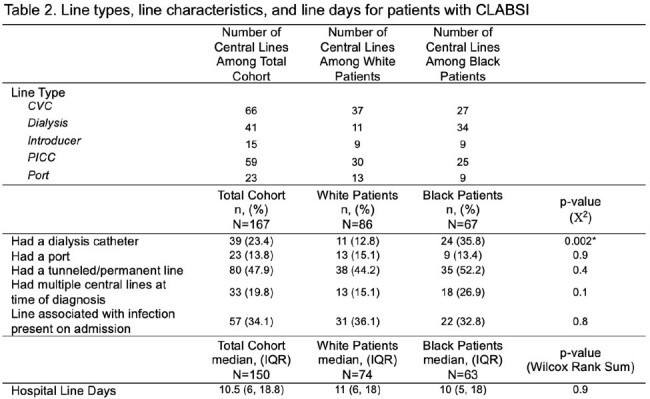

**Results:**

Among 167 patients with CLABSI, 86 (52%) identified as White and 67 (40%) identified as Black. Social and clinical characteristics of the cohort are included in Table 1. Data on central lines in place at diagnosis are included in Table 2. The 3 most common organisms were *Staphylococcus epidermidis*, *S. aureus*, and *Pseudomonas aeruginosa*. Median Elixhauser scores were not different between groups (9 v. 10, p = 0.2). Median Social Deprivation Index also did not differ between groups (64 v. 66, p = 0.4). Clinical and social characteristics identified via logistic regression as independently associated with race are described in Table 3.

**Figure:**
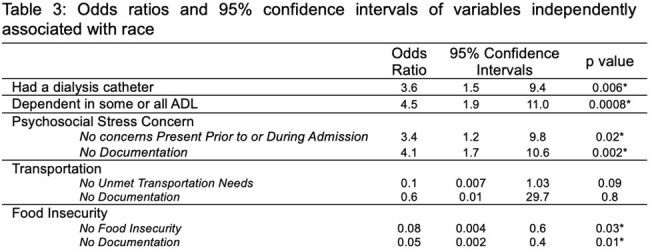

**Conclusion:**

Black patients with CLABSI more frequently required assistance in their ADLs, more frequently had a line dedicated to dialysis, and were less likely to have psychosocial concerns identified or documented. The results of this analysis support the complex interrelated nature of biopsychosocial factors that may predispose to disparate needs for central venous catheters and therefore inequities in the risk of CLABSI. Additionally, disparities in screening and documentation of SDOH exist in this cohort. Future studies should aim to quantify the risk of acquiring a CLABSI conferred by race.

**Disclosures:**

**Nicholas A. Turner, MD, MHSc**, PDI: Research contract|Purio Labs: Research contract

